# Serum vitamin D levels in non-obese women with polycystic ovary syndrome: a systematic review and meta-analysis

**DOI:** 10.3389/fendo.2026.1839319

**Published:** 2026-06-16

**Authors:** Mei Jiang, Tan Wang, Ling Huang

**Affiliations:** 1 Beijing Research Institute of Chinese Medicine, Beijing University of Chinese Medicine, Beijing, China; 2 School of Traditional Chinese Medicine, Beijing University of Chinese Medicine, Beijing, China

**Keywords:** 1,25-hydroxyvitamin D, 25-hydroxyvitamin D, meta-analysis, non-obese, polycystic ovary syndrome

## Abstract

**Introduction:**

Vitamin D deficiency is linked to increased risk of polycystic ovary syndrome (PCOS) and may worsen metabolic disorders such as insulin resistance. However, evidence remains inconsistent regarding vitamin D status in non−obese women with PCOS. This study aimed to perform the first systematic review and meta−analysis evaluating serum vitamin D levels in non−obese PCOS patients.

**Methods:**

Relevant studies were searched in PubMed, Embase, Cochrane Library, and Web of Science up to May 2025. Eligible studies comparing non−obese PCOS women and healthy controls were included. Two reviewers independently screened articles. Meta−analysis was conducted using a random−effects model, supplemented by subgroup, sensitivity, and publication bias analyses.

**Results:**

Eleven studies involving 533 non−obese PCOS patients and 574 controls were included. No significant differences were found in serum 25(OH)D or 1,25(OH)_2_D levels between groups. Heterogeneity was mainly attributed to region, diagnostic criteria, BMI, and assay methods. Significant publication bias was detected.

**Discussion:**

Serum vitamin D levels are not significantly associated with non−obese PCOS, implying vitamin D deficiency may be driven by obesity rather than PCOS pathogenesis. Vitamin D supplementation may benefit obese PCOS patients, and further prospective studies are needed.

**Systematic review registration:**

http://www.crd.york.ac.uk/PROSPERO, identifier CRD420251168257.

## Introduction

Polycystic ovary syndrome (PCOS), affecting 4%~21% of women worldwide, is a reproductive endocrine and metabolic disorder mainly characterized by (1) ovulatory dysfunction (infrequent or absent ovulation); (2) clinical and/or biochemical hyperandrogenism; and (3) polycystic ovary morphology (1). Clinically, it is mainly characterized by manifestations including menstrual disorders, infertility, hirsutism, and acne, with 60%~70% of patients being overweight or obese (1). PCOS is often accompanied by insulin resistance and abnormal glycolipid metabolism, which play a crucial role in its pathogenesis. This combination of metabolic abnormalities may increase the risk of complications such as diabetes, cardiovascular diseases, endometrial lesions, and mental/psychological disorders, causing serious adverse effects on patients’ reproductive, physical, and mental health (2).

Vitamin D is crucial for diverse physiological processes in the body, including the regulation of glucose metabolism and reproductive function, calcium homeostasis and bone mineralization, and immune modulation. Accumulating evidence indicates that vitamin D metabolism influences insulin and glucose function, suggesting a potential role for vitamin D in the development of insulin resistance and type 2 diabetes. In a large nationwide Danish primary care patient cohort, Rohold et al. (3) established an inverse association between serum 25-hydroxyvitamin D [25(OH)D] levels and type 2 diabetes risk. Another Mendelian randomization analysis provided suggestive evidence that genetically predicted lower serum 25(OH)D concentrations may causally increase the risk of type 2 diabetes and all-cause mortality (4). In diabetic rat models, vitamin D supplementation significantly reduces blood glucose levels (5). Meanwhile, studies demonstrate negative correlations of both body mass index (BMI) with vitamin D levels. Consistently, observational evidence indicates that serum 25(OH)D concentrations inversely associate with adiposity (6–8). In addition, Vitamin D plays a critical role in reproductive health and is critical for establishing and sustaining viable pregnancies. Vitamin D deficiency correlates with impaired fertility and increased risk of adverse pregnancy outcomes (9–11).

Evidence indicates vitamin D deficiency contributes to increased PCOS risk (12–14) and may exacerbate its characteristic metabolic derangements, including insulin resistance and metabolic syndrome (15–17). Furthermore, a growing body of evidence suggests that genetic variations in the vitamin D receptor (VDR) gene may also modulate PCOS susceptibility. An updated systematic review and meta-analysis by Heidarzadehpilehrood et al. demonstrated that women carrying ApaI, BsmI, Cdx2, and TaqI VDR gene polymorphisms have a significantly higher risk of PCOS, highlighting the genetic dimension of vitamin D signaling in PCOS pathogenesis (18). Although insulin resistance is central to PCOS pathogenesis, obesity exacerbates metabolic dysfunction primarily through intensifying insulin resistance and its downstream consequences. Thus, do non-obese women with PCOS exhibit reduced serum vitamin D concentrations compared with BMI-matched controls? The answer remains debated, with current evidence showing inconsistent conclusions (19–23). Therefore, this systematic review and meta-analysis aimed to compare serum vitamin D concentrations between non-obese women with PCOS and BMI-matched healthy controls.

## Materials and methods

### Reporting guidelines

This systematic review and meta-analysis adhered to the Preferred Reporting Items for Systematic Reviews and Meta-Analyses (PRISMA) guidelines and was prospectively registered with PROSPERO (CRD420251168257; http://www.crd.york.ac.uk/PROSPERO). Additionally, it followed the Meta-analysis of Observational Studies in Epidemiology (MOOSE) checklist.

### Search strategy

The PICO framework (Population, Intervention, Comparison, Outcome) was defined as follows: P (non-obese women), I (women with polycystic ovary syndrome), C (BMI-matched healthy women), O (serum vitamin D concentrations). An exhaustive literature search was conducted in PubMed, EMBASE, Cochrane Library, and Web of Science databases through May 2025 to identify relevant studies. Search terms included keywords related to these components without language, geographic, or journal restrictions: “polycystic ovary syndrome” OR “PCOS” AND “Non-obese” OR “nonobese” OR “normal weight” OR “normal-weight” AND “vitamin D” OR “25-hydroxyvitamin D” OR “25-OH-vitamin D” OR “25(OH)D” OR “vit D” OR “1, 25-hydroxyvitamin D” OR “1,25(OH)_2_D” OR “calcitriol” OR “cholecalciferol” OR “hydroxycholecalciferols” OR “calcifediol” OR “dihydroxycholecalciferols” OR “ergocalciferols”.

### Inclusion and exclusion criteria

Studies meeting all criteria were included: (1) original observational study of humans; (2) all subjects involved in studies were non-obese, defined as BMI < 30 kg/m² according to the World Health Organization (WHO) classification; (3) studies focusing on the association between serum vitamin D concentrations and PCOS; and (4) studies included data on serum vitamin D concentrations for patients with PCOS and BMI-matched healthy women.

Conference abstracts, meeting presentations, and congress proceedings were excluded. For duplicate publications from overlapping study populations, only the report with the largest sample size was retained; when sample sizes were equal, the most recent publication (by year) was chosen. If both were similar, the report with more detailed vitamin D measurements [e.g., both 25(OH)D and 1,25(OH)_2_D] was preferred.

### Study selection and data extraction

**Figure 1:**
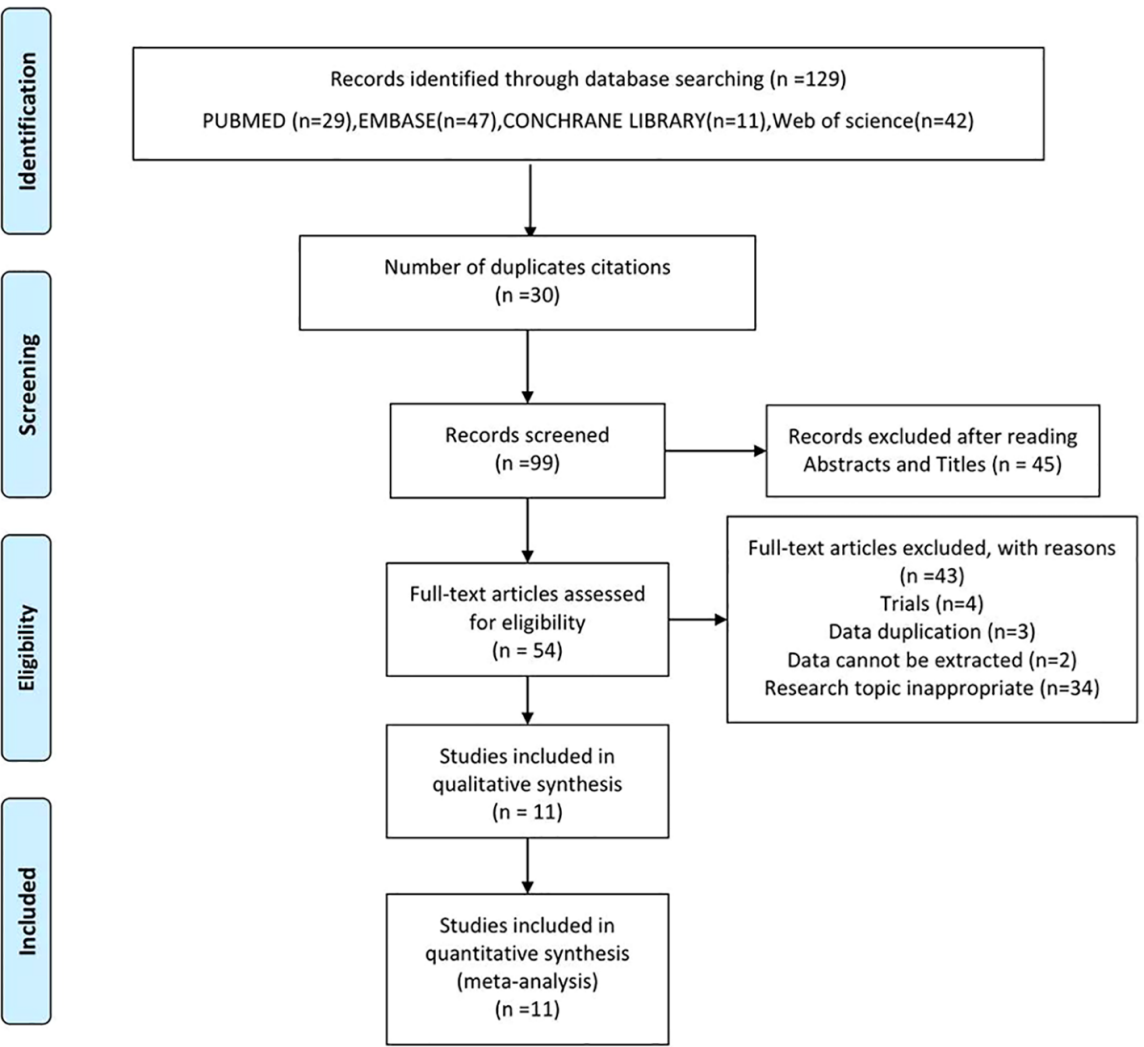
Flow chart of the selection process. From: Moher D, Liberati A, Tetzlaff J, Altman DG, The PRISMA Group (2009). Preferred Reporting Items for Systematic Reviews and Meta-Analyses: The PRISMA Statement. PLoS Med 6(7): e1000097. doi: 10.1371/journal.pmed1000097.

Two researchers (MJ and TW) independently performed literature screening and data extraction. Disagreements regarding eligibility or data interpretation were resolved through consultation with a third reviewer (LH). First, duplicates were identified and removed using EndNote. Subsequently, titles and abstracts were screened against inclusion criteria, excluding irrelevant studies. Finally, full texts were assessed with further exclusions based on predetermined criteria. A PRISMA-compliant flow diagram detailing the selection process was presented in **Figure 1**. Mean serum concentrations of 25-hydroxyvitamin D [25(OH)D] and 1,25-dihydroxyvitamin D [1,25(OH)_2_D] (± standard deviation [SD]) were extracted. When continuous variables were unreported, corresponding authors were contacted to request raw data. If contact attempts failed, SD values were imputed from the medians, quartiles or ranges using established methods (24, 25). All data were verified by LH.

### Quality assessment

Two reviewers independently assessed the methodological quality of included studies using the Newcastle-Ottawa Scale (NOS) system (26).

### Statistical analysis

Meta-analysis was performed on extracted data using pooled standardized mean differences (SMDs) with 95% confidence intervals (CIs) to account for heterogeneous vitamin D units. This assessed serum concentrations of 25(OH)D and 1,25(OH)_2_D in PCOS patients. Heterogeneity was quantified using Cochran’s Q test (two-sided) (27), with DerSimonian-Laird random-effects models applied when I-square (*I^2^*) ≥ 50%; otherwise, Mantel-Haenszel fixed-effect models were used (28, 29). Subgroup analyses assessed the relationships between serum 25(OH)D and 1,25(OH)_2_D concentrations across study characteristics, including geographical location, PCOS diagnostic criteria, BMI categories (≤25 kg/m² and >25 kg/m²), and vitamin D assay methods (RIA, ELISA/ECLIA, LC-MS/MS), to identify potential sources of heterogeneity. Obesity was defined as BMI ≥ 30 kg/m² according to the World Health Organization criteria. Sensitivity analyses tested result robustness by sequentially excluding individual studies. Funnel plots assessed publication bias when ≥ 10 studies were included. All meta-analyses employed RevMan software (version 5.3), with statistical significance defined as *P* < 0.05.

## Results

### Literature search

After screening titles and abstracts of 99 initially identified articles, 45 were excluded for not meeting inclusion criteria. Two reviewers independently evaluated the full text of the remaining 54 articles, from which 43 were excluded: 4 were clinical trials; 2 contained non-extractable data; 3 represented duplicate publications; and 34 were irrelevant to the research topic. Ultimately, 11 eligible observational studies were included (**Figure 1**), comprising individual data from 533 PCOS cases and 574 healthy controls (19–23, 30–35). **Table 1** summarizes the baseline characteristics of included studies, including author, publication year, geographical location, PCOS diagnosis criteria, sample size, age, BMI, measurement methods, and key findings.

**Table 1:** Summary characteristics of studies and participants.

No.	Study	Region	PCOS diagnosis criteria	Sample size (n) (cases vs. control)		Age(y) (cases vs. control)		BMI(kg/m^2^) (cases vs. control)		Measuring method of Vitamin D	Primary conclusion
				PCOS	Control	PCOS	Control	PCOS	Control		
1.	Panidis (2005)	Greece (Thessaloniki)	Rotterdam Criteria	169	70	22.82 ± 4.81	26.20 ± 6.11	21.54 ± 1.82	21.33 ± 1.84	RIA methods	Serum 25(OH)D increased and 1,25(OH)_2_D remained unchanged in PCOS
2.	Mahmoudi (2010)	Iran (Tehran)	the NIH criteria	29	49	28.52 ± 4.47	28.61 ± 6.02	23.06 ± 1.56	22.58 ± 2.80	RIA methods	Serum 25(OH)D remained unchanged and 1,25(OH)_2_D decreased in PCOS
3.	Yılmaz (2015)	Turkey (Konya)	Rotterdam Criteria	48	56	22.6 ± 2.9	23.5 ± 4.4	19-24.99	19-24.99	LC-MS/MS	Serum 25(OH)D decreased in PCOS
4.	Kuliczkowska-Plaksej (2019)	Poland (Wroclaw)	Rotterdam Criteria	77	55	25.5 ± 3.3	30.8 ± 4.54	21.4 ± 1.4	21.5 ± 1.4	RIA methods	Serum 25(OH)D remained unchanged in PCOS
5.	Aldhafiri (2023)	Saudi Arabia (Al-Madinah)	Rotterdam Criteria	30	100	30.97 ± 5.64	30.07 ± 5.31	23.23 ± 1.65	24.76 ± 3.00	RIA methods	Serum 25(OH)D decreased in PCOS
6.	Hegazy (2023)	Egypt (Cairo)	Rotterdam Criteria	20	17	24 ± 3.04	28 ± 6.18	24 ± 3.58	25 ± 2.72	ELISA	Serum 25(OH)D decreased in PCOS
7.	Hegde (2022)	India (Karnataka)	Rotterdam Criteria	102	139	24.71 ± 5.07	23.89 ± 6.24	<24.9	<24.9	ELISA	Serum 25(OH)D remained unchanged in PCOS
8.	Daghestani (2018)	Saudi Arabia (Riyadh)	Rotterdam Criteria	44	44	24.05 ± 4.41	25.43 ± 4.80	22.84 ± 1.52	22.12 ± 1.48	ELISA	Serum 25(OH)D remained unchanged in PCOS
9.	Cunningham (2019)	United Kingdom (Hull)	Rotterdam Criteria	29	30	30.9 ± 4.8	32.6 ± 4.7	26.0 ± 3.8	25.5 ± 3.6	LC-MS/MS	Serum 25(OH)D remained unchanged in PCOS
10.	Ozyurt (2022)	Turkey (Istanbul)	Rotterdam Criteria	30	30	26.9 ± 0.11	28.1 ± 1.04	24.8 ± 1.50	24.1 ± 0.33	ECLIA	Serum 25(OH)D remained unchanged in PCOS
11.	Butler (2024)	United Kingdom (Hull)	Rotterdam Criteria	29	30	30.7 ± 4.6	32.9 ± 4.6	25.9 ± 2.8	25.6 ± 2.7	LC-MS/MS	Serum 25(OH)D remained unchanged in PCOS

BMI, body mass index; 25(OH)D, 25-hydroxyvitamin D; 1,25(OH)_2_D, 1,25-hydroxyvitamin D; PCOS, polycystic ovary syndrome; RIA, Radioimmunoassay; ELISA, Enzyme-Linked Immunosorbent Assay; LC-MS/MS, Liquid Chromatography-Tandem Mass Spectrometry; NIH, National Institute of Child Health and Human Development; ECLIA, enzyme chemiluminescence immunoassay.

### Quality assessment

Two reviewers independently assessed study quality using the NOS system, with all included studies scoring ≥ 7 (**Table 2**).

**Table 2:** The results of NOS star system scoring.

No.	Study	Adequacy of case definition	Representativeness of cases	Selection of controls	Definition of controls	Comparability of cases and controls	Ascertainment of exposure	Same method of ascertainment for cases and controls	Non-Response rate	Total
1.	Panidis (2005)	1	1	0	1	2	1	1	0	7
2.	Mahmoudi (2010)	1	1	1	1	2	1	1	1	9
3.	Yılmaz (2015)	1	1	0	0	2	1	1	1	7
4.	Kuliczkowska-Plaksej (2019)	1	1	0	1	2	1	1	1	8
5.	Aldhafiri (2023)	1	1	0	1	2	1	1	1	8
6.	Hegazy (2023)	1	1	0	1	2	1	1	1	8
7.	Hegde (2022)	1	1	0	1	2	1	1	1	8
8.	Daghestani (2018)	1	1	0	1	2	1	1	1	8
9.	Cunningham (2019)	1	1	0	1	2	1	1	1	8
10.	Ozyurt (2022)	1	0	0	1	2	1	1	1	7
11.	Butler (2024)	1	1	0	1	2	1	1	1	8

### Meta-analysis results

**Figure 2:**
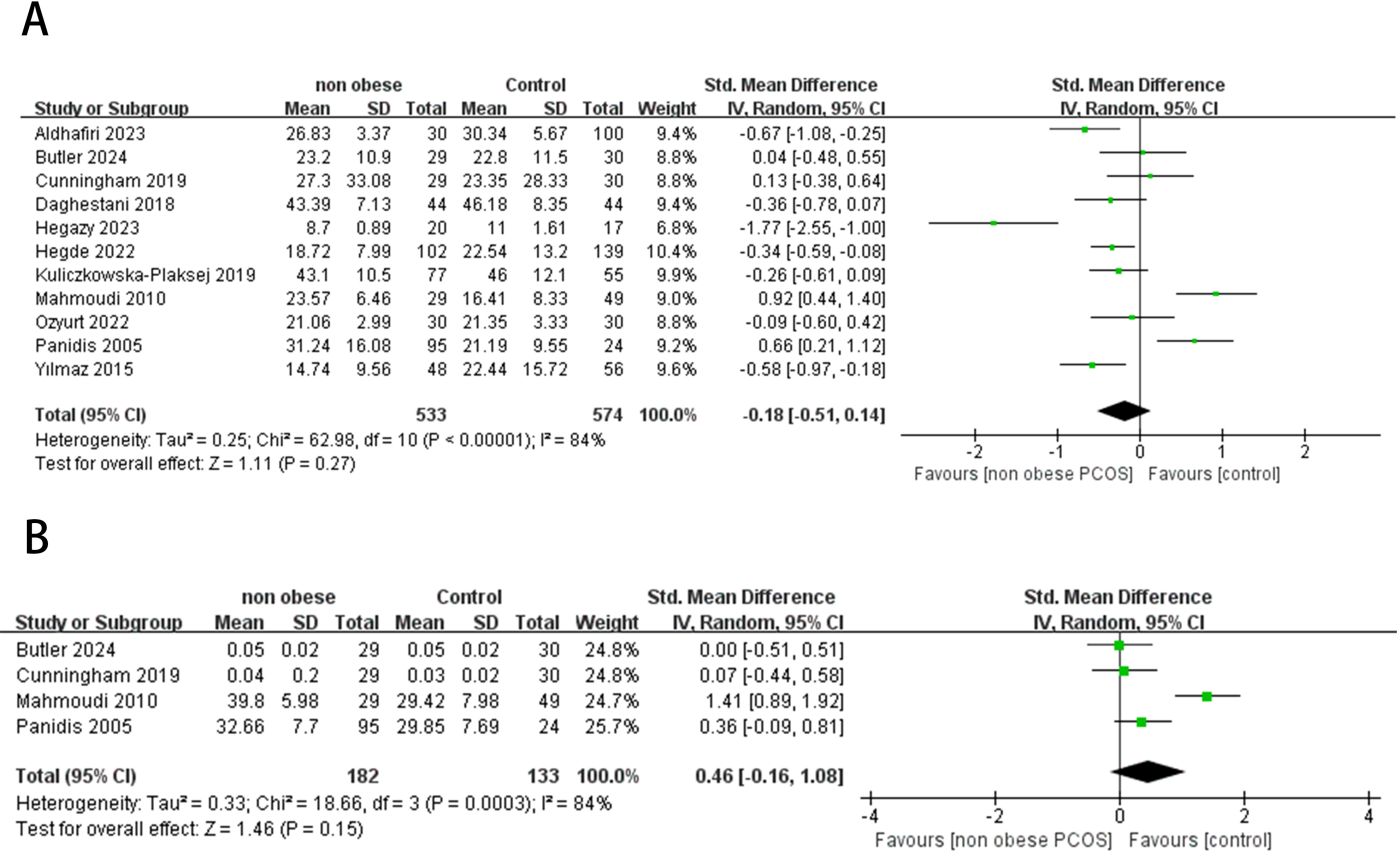
Forest plot of the levels of serum vitamin D concentrations in cases and healthy controls. Weights are from random effects analysis. **(A)** Meta-analysis of serum 25-hydroxyvitamin D concentrations. **(B)** serum 1,25-hydroxyvitamin D concentrations. CI, confidence interval; SD, standard difference.

Significant inter-study heterogeneity (*I^2^* = 84%, *P* < 0.00001) was observed in eleven studies (*n* = 1,107) comparing serum 25(OH)D concentrations between non-obese women with PCOS and healthy controls (**Figure 2A**). Pooled analysis demonstrated no significant inter-group difference in serum 25(OH)D concentrations (SMD: -0.18; 95% CI: -0.51 to 0.14; *P=*0.27).

Four studies (*n* = 315 participants) compared serum 1,25(OH)_2_D concentrations between non-obese women with PCOS and healthy controls (**Figure 2B**). Significant inter-study heterogeneity was observed (*I^2^* = 84%; *P* = 0.0003), and pooled analysis revealed no significant difference in serum 1,25(OH)_2_D concentrations between groups (SMD: 0.46; 95% CI: -0.16 to 1.08; *P=*0.15).

### Results of subgroup analysis

Substantial heterogeneity (*I²* > 50%) was detected across studies in this meta-analysis of serum vitamin D levels in PCOS patients and healthy controls. For heterogeneity exploration and enhanced accuracy in comparing non-obese PCOS women with healthy controls, predefined subgroup analyses were conducted with stratification based on geographical location, PCOS diagnosis criteria, BMI categories, and measuring methods.

Regional stratification (**Table 3**) revealed significantly lower serum 25(OH)D concentrations in African women with PCOS versus healthy controls (*P* < 0.00001). In contrast, European (*P* = 0.61) and Asian (*P* = 0.36) subgroups showed no significant differences, with reduced heterogeneity observed in the European cohort (*I^2^* = 61%).

**Table 3:** Effect estimate and heterogeneity of subgroup analysis for serum 25(OH)D concentrations.

Subgroup	Trials (n)	Sample size (n)	Effect estimate SMD (95% CI)	*I^2^*	*P*
Geographical location					
Europe	5	429	0.09 (-0.25, 0.42)	61%	0.61
Asia	5	641	-0.22(-0.68, 0.25)	86%	0.36
Africa	1	37	-1.77(-2.55, -1.00)	--	<0.00001
PCOS diagnosis criteria					
Rotterdam Criteria	10	1029	-0.29 (-0.57, 0.00)	78%	0.05
NIH Criteria	1	78	0.92 (0.44, 1.40)	--	0.0002
BMI categories (kg/m^2^)					
≤25	9	989	-0.24 (-0.62, 0.14)	87%	0.21
>25	2	118	0.08 (-0.28, 0.44)	0%	0.66
Measuring method of vitamin D					
RIA	4	459	0.16 (-0.55, 0.86)	91%	0.67
ELISA/ECLIA	4	426	-0.54 (-1.02, -0.06)	78%	0.03
LC-MS/MS	3	222	-0.16 (-0.63, 0.30)	66%	0.49

25(OH)D, 25-hydroxyvitamin D; CI, confidence interval; SMD, standard mean difference; PCOS, polycystic ovary syndrome; NIH, National Institute of Child Health and Human Development; BMI, body mass index; RIA, Radioimmunoassay; ELISA, Enzyme-Linked Immunosorbent Assay; ECLIA, enzyme chemiluminescence immunoassay; LC-MS/MS, Liquid Chromatography-Tandem Mass Spectrometry.

When stratified by PCOS diagnosis criteria (**Table 3**), women diagnosed by the National Institute of Child Health and Human Development (NICHD) criteria showed significantly higher serum 25(OH)D concentrations than healthy controls (*P* = 0.0002). In contrast, those diagnosed by Rotterdam criteria exhibited no significant difference in 25(OH)D concentrations compared with controls (*P* = 0.05).

Regarding BMI categories (**Table 3**), no significant difference in serum 25(OH)D concentrations was observed in either the BMI ≤ 25 kg/m^2^ (*P* = 0.21) or BMI > 25 kg/m^2^ (*P* = 0.66) subgroup. Notably, no heterogeneity was detected in the BMI >25 kg/m^2^ subgroup (*I^2^* = 0%).

When stratified by assay methods (**Table 3**), women with PCOS had significantly lower serum 25(OH)D concentrations than healthy controls in the ELISA/ECLIA subgroup (*P* = 0.03). In contrast, no significant difference was observed in either the Radioimmunoassay (RIA) (*P* = 0.67) subgroup or Liquid Chromatography-Tandem Mass Spectrometry (LC-MS/MS) (*P* = 0.49) subgroup. Notably, heterogeneity decreased substantially in the LC-MS/MS subgroup (*I^2^* = 66%).

### Sensitivity analysis

Sensitivity analysis was conducted using sequential exclusion of individual studies to evaluate result robustness. For serum 25(OH)D concentrations, the pooled effect size remained qualitatively unchanged after each exclusion, indicating robust meta-analysis outcomes (36). In contrast, serum 1,25(OH)_2_D analysis showed limited stability compared to Mahmoudi et al. (32). Exclusion of the latter study eliminated heterogeneity (*I^2^* = 0%).

### Publication bias

**Figure 3:**
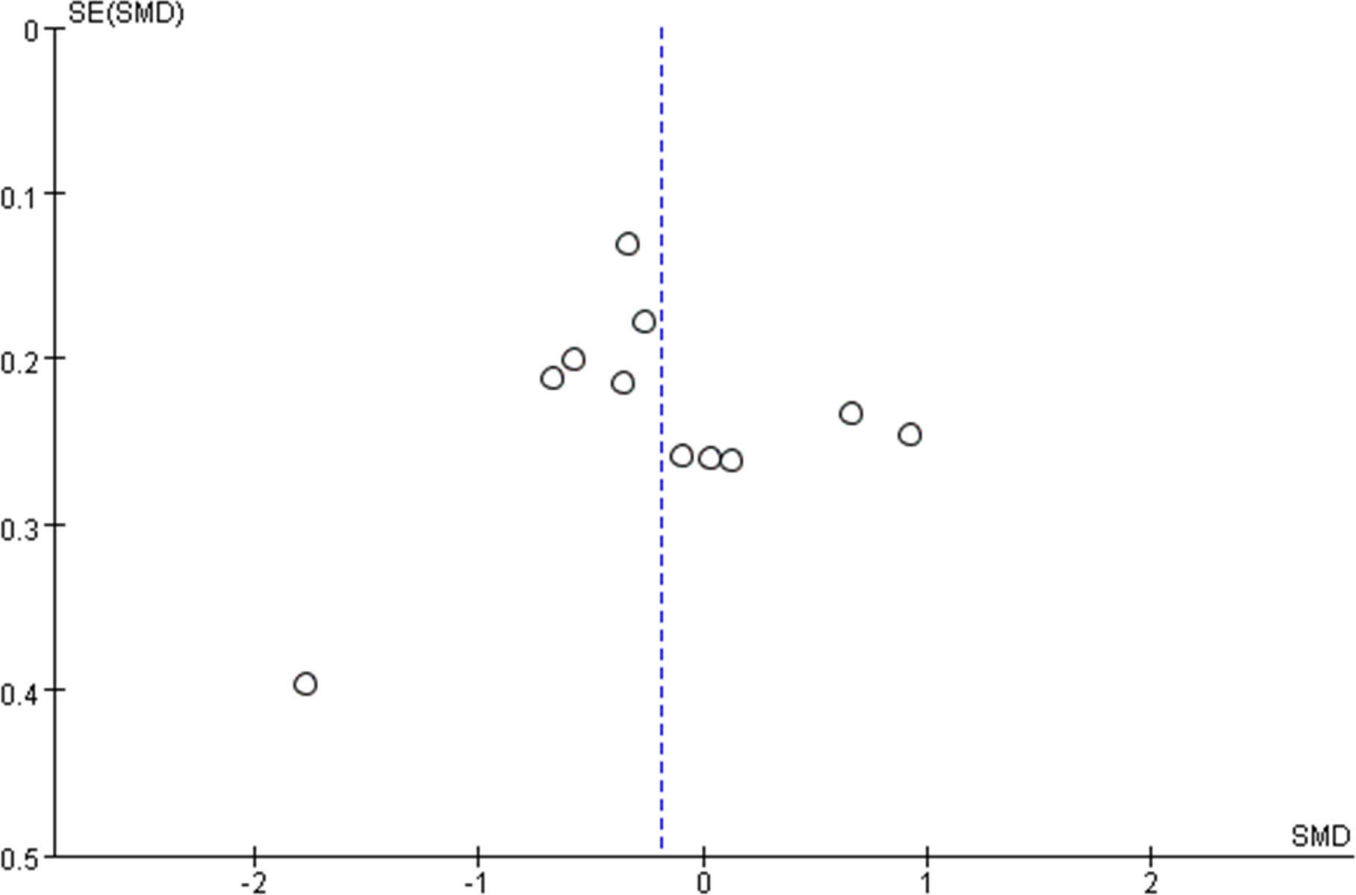
Funnel plot analysis of serum 25-hydroxyvitamin D concentrations.

Systematic reviewers risk introducing publication bias if their conclusions deviate from the evidence by excluding unpublished literature. For serum 25(OH)D concentrations, funnel plot asymmetry suggested potential publication bias (**Figure 3**). However, publication bias assessment regarding 1,25(OH)_2_D concentrations was omitted because the Cochrane Handbook for Systematic Reviews of Interventions (available at https://training.cochrane.org/handbook) states that meta-analyses with <10 studies lack sufficient statistical power for robust bias detection.

## Discussion

Although vitamin D deficiency is a well-established risk factor for PCOS pathogenesis (12–14), current evidence regarding its role in PCOS development among non-obese women remains inconclusive and merits rigorous validation. To our knowledge, this constitutes the first systematic review and meta-analysis quantifying serum 25(OH)D concentrations specifically in non-obese women with PCOS, encompassing 11 studies with 1,107 participants. Subgroup analyses were stratified by geographical region, PCOS diagnostic criteria, BMI categories, and measuring methods. The results demonstrated no significant difference in serum concentrations of either 25(OH)D or 1,25(OH)_2_D between non-obese women with PCOS and BMI-matched healthy controls.

Vitamin D, a fat-soluble micronutrient, is primarily synthesized in the skin through solar ultraviolet B (UVB) exposure. Paradoxically, despite greater skin surface area for sunlight absorption, obese individuals exhibit higher rates of vitamin D deficiency. Key mechanisms underlying this paradox include: the lipophilic nature of vitamin D drives its sequestration within hypertrophied adipocytes, significantly expanding its volume of distribution and diluting circulating concentrations despite potentially adequate total body stores; metabolic dysregulation further compromises vitamin D status: hepatic 25-hydroxylation (mediated primarily by CYP2R1) may be impaired by non-alcoholic fatty liver disease, reducing conversion to 25(OH)D; renal activation to the hormonal form 1,25(OH)D via CYP27B1 is suppressed due to obesity-associated endocrine disturbances - including decreased parathyroid hormone (PTH) secretion (from adipose tissue-mediated calcium buffering), elevated fibroblast growth factor 23 (FGF23), and chronic low-grade inflammation. This proinflammatory milieu, characterized by elevated cytokine levels and dysregulated adipokines, also upregulates the catabolic enzyme 24-hydroxylase, accelerating vitamin D metabolite degradation; concomitant behavioral factors (reduced UVB exposure, dietary inadequacy) exacerbate deficiency. Moreover, adipose tissue inflammation induces functional vitamin D resistance by downregulating expression of the vitamin D receptor (VDR), a nuclear transcription factor, and by impairing its signaling (37).

Vitamin D exerts its biological effects by binding to the VDR, which is a ligand-dependent transcription factor widely expressed in both male and female germ cells. This receptor-mediated mechanism qualifies vitamin D as a steroid hormone. Studies have confirmed VDR expression throughout the female reproductive tract, including ovarian tissue, fallopian tubes, and endometrium. Vitamin D mediates critical fertility-related functions through VDR binding that regulates both ovarian physiology and immunomodulatory pathways (38–40). Accumulating evidence from the past decade indicates that vitamin D deficiency is associated with impaired fertility in both sexes (39). Vitamin D deficiency (VDD), defined as serum 25(OH)D < 20 ng/mL, affects 67%~85% of women with PCOS (41). A meta-analysis confirms significantly lower serum 25(OH)D levels in PCOS patients versus healthy controls, with further reductions observed in obese PCOS subgroups (41). Consistent with this pattern, our results demonstrated comparable vitamin D levels between non-obese women with PCOS and healthy controls. Mechanistically, high VDR density in adipocytes promotes sequestration of lipid-soluble vitamin D in adipose tissue, impairing its conversion to bioactive form. Adiposity thus emerges as the primary driver underlying VDD in PCOS patients.

Observational studies linking low serum 25(OH)D concentrations to ovulatory dysfunction, menstrual irregularities, and hyperandrogenism suggest that VDD likely participates in the development of PCOS pathology (42–44). Hyperandrogenism constitutes a core diagnostic criterion for PCOS, assessed via serum measurements of total testosterone (TT), dehydroepiandrosterone sulfate (DHEAS), and sex hormone-binding globulin (SHBG), with subsequent calculation of free androgen index (FAI). Current evidence demonstrates bidirectional associations between VDD and hyperandrogenism markers (14): specifically, a positive correlation exists between vitamin D concentrations and SHBG levels, while inverse relationships are observed with TT, DHEAS, and FAI (45, 46). Notably, PCOS women with VDD exhibit heightened hyperandrogenemia risk among those aged < 26 years with overweight (47). Supporting this metabolic interplay, Palm et al. reported that 3-month vitamin D supplementation reduced TT and androstenedione levels without altering insulin resistance parameters in obese PCOS patients (48). Further study revealed significantly lower vitamin D levels in hyperandrogenemic versus non-hyperandrogenemic women—a finding compounded by high obesity prevalence (67.2%) in this subgroup, suggesting adipose-mediated sequestration may contribute to vitamin D depletion (49).

Insulin resistance is most prevalent in PCOS patients with obesity (65%), but also occurs in approximately 20% of those without obesity (50). In women with PCOS and normal glucose tolerance, the annual conversion rate to impaired glucose tolerance is 16% (51). Beyond obesity and intrinsic PCOS pathology, additional risk factors contribute to the high prevalence of glucose metabolism disorders and insulin resistance in this population. Notably, 25(OH)D deficiency constitutes an established risk factor for insulin resistance and diabetes. Experimentally, oral administration of 1,25(OH)D_3_ protects non-obese diabetic mice from insulin-dependent diabetes mellitus (52) and ameliorates streptozotocin-induced diabetes in diabetic rats (53). Vitamin D deficiency exacerbates phenotypic features of PCOS and is independently associated with adverse cardiovascular risk markers (54). Our results are corroborated by evidence showing reduced 25(OH)D in obese women regardless of PCOS status, relative to non-obese PCOS individuals (55). Notably, a study reported significantly lower serum 25(OH)D levels in PCOS patients with elevated cardiovascular risk compared to those without, highlighting vitamin D’s pathophysiological role in cardiometabolic disturbances associated with PCOS (56). Thus, vitamin D supplementation could be a component of the complex management strategy for women with PCOS and obesity who present with vitamin D deficiency, aimed not only at ameliorating insulin resistance but also at preventing other serious long-term comorbidities.

The NOS system is a key criterion for assessing the risk of bias in studies included in systematic reviews, directly influencing the level of evidence. Using the NOS scale to evaluate literature quality, all selected studies scored ≥7 points, indicating a low risk of bias in the included studies in this meta-analysis. Subgroup analyses were performed to investigate underlying heterogeneity causes and to rigorously evaluate the association between vitamin D levels and PCOS in non-obese women. Heterogeneity in this meta-analysis of serum 25(OH)D-PCOS correlation may arise from geographic variations, diagnostic criteria discrepancies, BMI stratification, and methodological differences in vitamin D assays. (1) Regional variations in dietary patterns, lifestyle behaviors, and socioeconomic status significantly influence vitamin D status; (2) The NICHD criteria diagnostic framework imposes more stringent criteria than the Rotterdam consensus, whereas the latter encompasses a broader patient spectrum; (3) Literature shows varying BMI thresholds for defining non-obese status: while most studies use BMI < 25 kg/m², a minority employ BMI < 29 kg/m². This inconsistency positions BMI as a potential source of heterogeneity; (4) Different assay methodologies for serum 25(OH)D measurement exhibit distinct measurement variability, making analytical techniques a likely source of heterogeneity. In addition to these identified sources, other unmeasured or unreported factors might have contributed to the residual heterogeneity. For instance, seasonal variation in blood collection could influence serum 25(OH)D levels due to differences in sunlight exposure, but none of the included studies reported the season of sampling. Similarly, skin phototype, which affects cutaneous vitamin D synthesis, was not documented in the original studies. While geographic region (e.g., Africa, Asia, Europe) may partly capture differences in sunlight intensity and lifestyle, individual−level data on sun exposure habits, dietary vitamin D intake, or supplement use were unavailable, precluding further adjustment. Future primary studies should record these variables to enable more precise heterogeneity exploration.

Although subgroup analyses identified specific sources of heterogeneity, significant residual heterogeneity persisted, necessitating sensitivity analyses. For serum 25(OH)D concentrations, the pooled effect size demonstrated robustness, remaining directionally consistent after sequential study exclusion. Conversely, the meta-analysis of serum 1,25(OH)_2_D concentrations exhibited significant sensitivity to Mahmoudi et al.’s study (32). Upon its exclusion, heterogeneity markedly decreased (*I^2^* decreased from 84% to 0%), indicating that this study substantially contributed to between-study heterogeneity. *Post hoc* examination revealed that the inclusion criteria for PCOS in this study was the NICHD criteria, while the inclusion criteria in other studies were the Rotterdam criteria. Hence, the inconsistency in inclusion criteria may have contributed to the heterogeneity. In addition, this meta-analysis shows possible signs of publication bias. Publication bias denotes the systematic tendency for positive or statistically significant findings to be published more readily than null results in peer-reviewed journals. Therefore, careful consideration is required prior to clinical implementation of these findings.

Nonetheless, we acknowledge this meta-analysis has limitations: (1) substantial heterogeneity existed across included studies, primarily attributable to geographical variations in participant recruitment, diagnostic criteria, BMI stratification, methodological variations in vitamin D assays, and higher vitamin D supplement usage rates in developed countries. Differences between studies could stem from both heterogeneous population distributions and methodological limitations. And the funnel plot asymmetry for 25(OH)D suggests the presence of publication bias. Publication bias refers to the tendency for positive or statistically significant findings to be published more readily than null or negative results. In the context of our meta-analysis, if smaller studies with lower vitamin D levels in PCOS patients remained unpublished, the pooled effect size might be biased toward the null (i.e., no difference). However, it is also possible that the asymmetry arose from other factors, such as true between−study heterogeneity (e.g., variations in study quality or design) rather than publication bias per se. Nonetheless, the existence of publication bias implies that our conclusion of “no significant association” should be interpreted cautiously. The true effect could be either a small positive or a small negative association that current published evidence fails to capture. Therefore, our findings do not definitively rule out a clinically meaningful difference, but rather suggest that if such a difference exists, it is likely small and may be masked by the absence of unpublished null studies. Registration of observational study protocols and publication of results regardless of the direction of effect would help mitigate this bias in the future; (2) the main confounding factors affecting patients’ vitamin D levels in the original studies (e.g., ethnicity, physical activity, sun exposure habits, diet, and daily calcium intake) were not adjusted for. This might affect the conclusions of our meta-analysis. Therefore, further prospective cohort studies adjusting for these confounders are warranted to validate these findings; (3) the small number of included studies, combined with their predominantly case-control designs, limits causal inferences about whether VDD causes PCOS or results from it. Therefore, prospective cohort studies are warranted to characterize the evolution of VDDover time in obese women with PCOS. Consequently, these results warrant cautious interpretation.

Furthermore, longitudinal prospective studies are warranted to track serum vitamin D levels in non−obese women with PCOS who subsequently gain weight. Such studies could help determine whether weight gain precipitates a decline in vitamin D status in this population and whether this decline is independent of PCOS−related metabolic changes. This would provide stronger causal evidence for the hypothesis that obesity, rather than PCOS *per se*, drives vitamin D deficiency.

## Conclusion

In summary, this systematic review and meta-analysis found no significant association between serum vitamin D concentrations and PCOS in non-obese women. This implies that serum vitamin D levels depend more on body weight than on PCOS status, and vitamin D deficiency may therefore be attributable to obesity rather than directly linked to PCOS *per se*. Consequently, vitamin D deficiency should not be interpreted as an independent pathogenic factor for metabolic disorders in PCOS. Prospective studies evaluating the therapeutic efficacy of vitamin D supplementation in obese women with PCOS are nevertheless warranted.

## Data Availability

The original contributions presented in the study are included in the article/**Supplementary Material**. Further inquiries can be directed to the corresponding author.
