# Supplementary material for: Serum vitamin D levels in non-obese women with polycystic ovary syndrome: a systematic review and meta-analysis
Source: Front Endocrinol (Lausanne). 2026 Jun 16;17:1839319. doi: 10.3389/fendo.2026.1839319 (PMC13314477; doi:10.3389/fendo.2026.1839319)
Supplement: Supplementary file 1 [file DataSheet1.doc]

**MOOSE Checklist**

**Serum Vitamin D Levels in Non-obese Women with Polycystic Ovary Syndrome: A Systematic Review and Meta-Analysis**

Mei Jiang^a，†^, Tan Wang^b，†^, Ling Huang^b*^

^a^ Beijing Research Institute of Chinese Medicine, Beijing University of Chinese Medicine, Beijing;

^b^ School of Traditional Chinese Medicine, Beijing University of Chinese Medicine, Beijing.

^†^These authors contributed equally to this work.

***Correspondence:**Ling Huang, School of Traditional Chinese Medicine, Beijing University of Chinese Medicine, No.11 Beisanhuandong Road, Chaoyang District, 100029, Beijing, P. R. China. E-mail: lhwang@bucm.edu.cn.

| **Criteria** | | **Brief description of how the criteria were handled in the meta-analysis** |
| --- | --- | --- |
| **Reporting of background should include** | |  |
| √ | Problem definition | Many studies have found that vitamin D deficiency is associated with an elevated risk of polycystic ovary syndrome (PCOS) development and may exacerbate its characteristic metabolic derangements, including insulin resistance and metabolic syndrome. However, studies report inconsistent results on vitamin D's role in PCOS development among non-obese women. |
| √ | Hypothesis statement | Serum vitamin D levels did not differ between non-obese women with PCOS and healthy controls. |
| √ | Description of study outcomes | Serum vitamin D concentrations |
| √ | Type of exposure or intervention used | Non-obese women with PCOS |
| √ | Type of study designs used | We included original human observational studies; We excluded experimental research or animal studies. |
| √ | Study population | Non-obese women with PCOS and BMI-matched healthy controls |
| **Reporting of search strategy should include** | |  |
| √ | Qualifications of searchers | None. |
| √ | Search strategy, including time period included in the synthesis and keywords | Through May 2025 to identify published studies using the following keywords: “polycystic ovary syndrome” OR “PCOS” AND “Non-obese” OR “nonobese” OR “normal weight” OR “normal-weight” AND “vitamin D” OR “25-hydroxyvitamin D” OR “25-OH-vitamin D” OR “25(OH)D” OR “vit D” OR “1, 25-hydroxyvitamin D” OR “calcitriol” OR “cholecalciferol” OR “hydroxycholecalciferols” OR “calcifediol” OR “dihydroxycholecalciferols” OR “ergocalciferols” |
| √ | Databases and registries searched | The Cochrane Library, PubMed, EMBASE, and Web of Science databases; the PROSPERO database (http://www.crd.york.ac.uk/PROSPERO) |
| √ | Search software used, name and version, including special features | We did not employ a search software. EndNote was used to merge retrieved citations and eliminate duplications. |
| √ | Use of hand searching | We hand-searched bibliographies of retrieved papers for additional references. |
| √ | List of citations located and those excluded, including justifications | Details of the literature search process are outlined in the flow chart. The citation list is available upon request. |
| √ | Method of addressing articles published in languages other than English | We placed no restrictions on language; there were no studies outside of English. |
| √ | Method of handling abstracts and unpublished studies | We had contacted a few authors for unpublished studies on the association. |
| √ | Description of any contact with authors | We contacted authors who had reported outcome indicator, but had not reported specific data. |
| **Reporting of methods should include** | |  |
| √ | Description of relevance or appropriateness of studies assembled for assessing the hypothesis to be tested | Detailed inclusion and exclusion criteria were described in the methods section. |
| √ | Rationale for the selection and coding of data | Data extracted from each of the studies were relevant to the population characteristics, study design, exposure, outcome, and possible effect modifiers of the association. |
| √ | Assessment of confounding | The main confounding factors, such as ethnicity, physical activity, sun exposure habits, diet, and daily calcium intake, affecting serum vitamin D concentrations in the original study were not adjusted, which had been reported as a limitation in the discussion. |
| √ | Assessment of study quality, including blinding of quality assessors; stratification or regression on possible predictors of study results | Subgroup analyses were conducted according to geographical location, PCOS diagnosis criteria, BMI categories, and measuring methods. |
| √ | Assessment of heterogeneity | Heterogeneity of the studies were explored within two types of study designs using Cochrane’s Q test of heterogeneity and *I^2^* statistic that provides the relative amount of variance of the summary effect due to the between-study heterogeneity. |
| √ | Description of statistical methods in sufficient detail to be replicated | Description of methods of meta-analyses, sensitivity analyses, and assessment of publication bias are detailed in the methods. |
| √ | Provision of appropriate tables and graphics | We included 1 box detailing the terms used for database search, 1 flow chart,1 summary table, 1 forest plot of all studies, 1 table of subgroup analyses, 1 funnel plot of serum 25(OH)D concentrations. |
| **Reporting of results should include** | |  |
| √ | Graph summarizing individual study estimates and overall estimate | Figure 2 |
| √ | Table giving descriptive information for each study included | Table 1 |
| √ | Results of sensitivity testing | For serum 25(OH)D concentrations, the pooled effect size remained qualitatively unchanged after each exclusion, indicating robust meta-analysis outcomes. In contrast, serum 1,25(OH)₂D analysis showed limited stability compared to Mahmoudi et al. |
| √ | Indication of statistical uncertainty of findings | 95% confidence intervals were presented with all summary estimates, *I^2^* values and results of sensitivity analyses |
| **Reporting of discussion should include** | |  |
| √ | Quantitative assessment of bias | The quality assessment was according to the Newcastle-Ottawa Scale system. |
| √ | Justification for exclusion | The main confounding factors, such as ethnicity, physical activity, sun exposure habits, diet, and daily calcium intake, affecting serum vitamin D concentrations in the original study were not adjusted, which had been reported as a limitation in the discussion. |
| √ | Assessment of quality of included studies | The NOS system is a key criterion for assessing the risk of bias in studies included in systematic reviews, directly influencing the level of evidence. Using the NOS scale to evaluate literature quality, all selected studies scored ≥7 points, indicating a low risk of bias in the included studies in this meta-analysis. |
| **Reporting of conclusions should include** | |  |
| √ | Consideration of alternative explanations for observed results | We discussed that potential unmeasured confounders such as ethnicity, physical activity, sun exposure habits, diet, and daily calcium intake may have caused residual confounding, but the measured factors that are correlated with such confounders would have mitigated the bias. |
| √ | Generalization of the conclusions | We found that no significant association between serum vitamin D concentrations and PCOS in non-obese women. This suggests vitamin D deficiency is likely mediated by obesity rather than directly linked to PCOS pathogenesis. |
| √ | Guidelines for future research | Prospective studies investigating the therapeutic utility of vitamin D supplementation in obese PCOS patients are warranted |
| √ | Disclosure of funding source | This work was supported by the Fundamental Research Funds for the Central Universities (No. 2025-JYB-KYPT-04). |
