# Supplementary material for: Serum vitamin D levels in non-obese women with polycystic ovary syndrome: a systematic review and meta-analysis
Source: Front Endocrinol (Lausanne). 2026 Jun 16;17:1839319. doi: 10.3389/fendo.2026.1839319 (PMC13314477; doi:10.3389/fendo.2026.1839319)
Supplement: Supplementary file 2 [file DataSheet2.docx]

**Supplemental Table Ⅰ** Search strategy and results for PubMed

| **Search strategy** | | **Results** |
| --- | --- | --- |
| #1 | Polycystic Ovary Syndrome[Mesh] | 19873 |
| #2 | PCOS[Title/Abstract] OR polycystic ovary syndrome[Title/Abstract] | 22513 |
| #3 | #1 OR #2 | 26493 |
| #4 | Non-obese or nonobese or normal weight or normal-weight | 49957 |
| #5 | Vitamin D[Mesh] | 72360 |
| #6 | 25-Hydroxyvitamin D 2[Mesh] OR Calcifediol[Mesh] | 5269 |
| #7 | 25-Hydroxyvitamin D3 1-alpha-Hydroxylase[Mesh] OR Calcitriol[Mesh] | 15981 |
| #8 | Cholecalciferol[Mesh] | 29842 |
| #9 | Hydroxycholecalciferols[Mesh] | 22557 |
| #10 | Dihydroxycholecalciferols[Mesh] | 16736 |
| #11 | Ergocalciferols[Mesh] | 4618 |
| #12 | 25-hydroxyvitamin D[Title/Abstract] OR 25-OH-vitamin D[Title/Abstract] OR 25(OH)D[Title/Abstract] OR vit D[Title/Abstract] OR vitamin D[Title/Abstract] OR 1, 25-hydroxyvitamin D[Title/Abstract] OR calcitriol[Title/Abstract] OR Cholecalciferol[Title/Abstract] OR Hydroxycholecalciferols[Title/Abstract] OR Calcifediol[Title/Abstract] OR Dihydroxycholecalciferols[Title/Abstract] OR Ergocalciferols[Title/Abstract] | 92233 |
| #13 | #5 OR #6 OR #7 OR #8 OR #9 OR #10 OR #11 OR #12 | 109614 |
| #14 | #3 AND #4 AND #13 | 29 |

**Supplemental Table Ⅱ** Search strategy and results for Cochrane Library

| **Search strategy** | | **Results** |
| --- | --- | --- |
| #1 | MeSH descriptor: [Polycystic Ovary Syndrome] explode all trees | 2144 |
| #2 | (PCOS):ti,ab,kw OR (polycystic ovary syndrome):ti,ab,kw | 5473 |
| #3 | #1 OR #2 | 5473 |
| #4 | (Non-obese or nonobese or normal weight or normal-weight):ti,ab,kw | 23405 |
| #5 | MeSH descriptor: [Vitamin D] explode all trees | 7588 |
| #6 | MeSH descriptor: [Calcitriol] explode all trees | 1101 |
| #7 | MeSH descriptor: [Cholecalciferol] explode all trees | 4805 |
| #8 | MeSH descriptor: [Hydroxycholecalciferols] explode all trees | 1823 |
| #9 | MeSH descriptor: [Dihydroxycholecalciferols] explode all trees | 1161 |
| #10 | MeSH descriptor: [Ergocalciferols] explode all trees | 1458 |
| #11 | (25(OH)D or vit D or vitamin D or calcitriol OR Cholecalciferol OR Hydroxycholecalciferols OR Calcifediol OR Dihydroxycholecalciferols OR Ergocalciferols):ti,ab,kw | 20445 |
| #12 | #5 OR #6 OR #7 OR #8 OR #9 OR #10 OR #11 | 20451 |
| #13 | #3 AND #4 AND #12 | 11 |

**Supplemental Table Ⅲ** Search strategy and results for Embase

| **Search strategy** | | **Results** |
| --- | --- | --- |
| #1 | (pcos OR polycystic) AND ('ovary'/exp OR ovary) AND syndrome:ab,ti | 33590 |
| #2 | 'ovary polycystic disease'/exp | 42690 |
| #3 | #1 OR #2 | 44946 |
| #4 | ('non obese' OR nonobese OR normal) AND ('weight'/exp OR weight) OR 'normal weight':ab,ti | 293979 |
| #5 | '25 hydroxyvitamin d'/exp | 31075 |
| #6 | 'vitamin d'/exp | 199445 |
| #7 | 'calcifediol'/exp | 11624 |
| #8 | 'calcitriol'/exp | 36734 |
| #9 | 'colecalciferol'/exp | 30223 |
| #10 | 'hydroxycolecalciferol'/exp | 942 |
| #11 | 'dihydroxycolecalciferol'/exp | 903 |
| #12 | 'ergocalciferol'/exp | 10729 |
| #13 | (((('25 hydroxyvitamin' AND d OR '25 oh vitamin') AND d OR vit) AND d OR vitamin) AND d OR 1,) AND '25 hydroxyvitamin' AND d OR calcitriol OR cholecalciferol OR hydroxycholecalciferols OR calcifediol OR dihydroxycholecalciferols OR ergocalciferols:ab,ti | 82030 |
| #14 | #5 OR #6 OR #7 OR #8 OR #9 OR #10 OR #11 OR #12 OR #13 | 202234 |
| #15 | #3 AND #4 AND #14 | 47 |

**Supplemental Table Ⅳ** Search strategy and results for Web of Science

| **Search strategy** | | **Results** |
| --- | --- | --- |
| #1 | TS=(PCOS or polycystic ovary syndrome) | 23939 |
| #2 | TS=(Non-obese or nonobese or normal weight or normal-weight) | 123742 |
| #3 | TS=(25-hydroxyvitamin D or 25-OH-vitamin D or 25(OH)D or vit D or vitamin D or 1, 25-hydroxyvitamin D or calcitriol OR Cholecalciferol OR Hydroxycholecalciferols OR Calcifediol OR Dihydroxycholecalciferols OR Ergocalciferols ) | 106489 |
| #4 | #1 AND #2 AND #3 | 42 |
